# A Case Report on Management of Father Daughter Incest with Schizophrenia

**DOI:** 10.1155/2016/4010187

**Published:** 2016-12-05

**Authors:** Tanjir Rashid Soron

**Affiliations:** Z H Shikder Women's Medical College, Dhaka, Bangladesh

## Abstract

Incest is a neglected and hidden public health problem. This case is about a patient who was victim of sexual abuse, suffered from schizophrenia and abused his biological daughter. He was physically and sexually abused by seniors and classmates, developed paranoid delusion and auditory hallucination. During the course of the illness, he was hospitalized several times as a case of schizophrenia and sexual dysfunction was his main concern. The patient's illness followed a waxing and waning course. He took medication on on-and-off basis. He abused his biological daughter sexually at the later stage of the illness. Ultimately, the patient attempted suicide after an indecent sexual act with another relative and he was admitted to the hospital. He was treated with risperidone that was titrated to 10 mg per day. After continuing the medication for 2 years he regained a functioning life and remained stable with medication. This case shows the importance of exploring the sexual behavior of the patients and sharing the experience may help in the treatment of schizophrenia patients with incest.

## 1. Introduction

Childhood abuse or maltreatment disrupts the smooth growth of mental health. It increases the risk of mood disorders, anxiety disorders, alcohol use disorders, drug use disorders, disruptive behavior disorders, antisocial behavior, and psychosis [1]. Among the various type of abuse, sexual abuse is most distressing and when the perpetrator is a family member; it is extremely unbearable, shameful, and painful. The evidence of childhood trauma in the etiology of schizophrenia is increasing [2]. However, the relation with incest and schizophrenia is yet to be explored in wide scale.

Incest is defined as any sexual activity between close blood relatives who are forbidden by law to marry [3]. It remains a hidden, neglected social and mental health issue. Furthermore, most of the families, societies, and states try to hide the incident to maintain their social status. However, the problem is increasing and the World Health Organization (2004) classifies incest as a silent health emergency considering its increasing incidence, importance, and impact [4]. Among the various types of incest, father-daughter incest is the most common, followed by the other types like brother-sister, sister-sister, and mother-son incest [5]. Father-daughter incest is more prevalent where parents involve in verbal or physical fighting or brutality, and they accept father-daughter nudity, lack of maternal affection, and being in a home headed by single-parent mother where divorce or death of the father had resulted in a man other than the biological father living in the home [6]. Evidence shows aggressive and sexually inappropriate behavior is common in schizophrenia with persisting psychotic symptoms and impaired decision-making capacity [7]. Victims of sexual abuse hardly report these incidents in the developing countries due to negative attitude and stigma towards the victim. This may be one of the important reason of rare reporting of incest. Even if the family came to know the indecent, they try to keep it secret [8].

People are shy and uncomfortable discussing sex in the South East Asia. Moreover, they have stigmatized negative attitude to mental illness and sexual problems. Society undermines and neglects the importance of healthy sexual life. Interestingly, in rural areas people consider marriage will cure the mental illness and they believe psychiatric treatment is ineffective in treating mental illness. Physicians are uncomfortable asking about sexual behavior of the patient due to social and cultural tradition. Hence asking question about incest is beyond imagination and there is no significant information regarding the management of problem along with other mental illnesses.

So far, to the knowledge of the author, this is the first article in the Indian subcontinent that focused on father-daughter incest in schizophrenia. This case focused on the necessity of evaluating the sexual behavior of schizophrenic patients and raised few questions regarding the role of childhood sexual abuse in sexual behavior. Another point raised in the article is the superiority of risperidone in management incestuous behavior in schizophrenia. This case will encourage the psychiatrist to evaluate the neglected area as well as treating such sensitive cases.

## 2. The Case

A thirty-one-year old married male was brought to the psychiatry service due to a suicide attempt after an indecent sexual behavior. He was perplexed, suspicious, and seemed talking with hallucinatory figure.

The patient was brought up in a conservative family and studied in a residential religious institution. His teachers and senior students abused him physically and sexually at the age of eight. Within a few months, this boy started abusing new students sexually and developed sexual relation with multiple boys. At the age of 16 years, his most intimate sexual partner left the hostel. He became suspicious that other students and teachers conspired against him and forced the partner to leave the hostel. Afterwards his suspiciousness spread to every aspect of his life. He refused to take food and drinks to avoid poisoning and loss of sexual power. Moreover, he started experiencing auditory hallucination (second and third persons) that centered on his poor sexual performance and paranoid delusion of poisoning. He lost his judgment and insight. His disorganized behavior of becoming nude in public places and masturbating warned the family members. He was admitted in the psychiatry hospital and was treated with haloperidol up to 30 mg initially by injectable route then orally. He had to discontinue his study due to his deteriorating mental state. After the discharge from the hospital he started going to brothels. The young man became frustrated and anxious due to lack of rigidity and ejaculation within 20–30 seconds most of the times. He failed to resist the temptation of touching the private parts of the females in the market, bus stoppage, and other crowded places. He was punished several times; however, the physical punishment by family members or by the angry mobs failed to prevent him from these types of act.

His family assumed that his symptoms might subside after marriage. The couple stayed with the parents and four siblings. However, his sexual problem continued throughout his conjugal life. His psychotic feature fluctuated; however, delusion of infidelity appeared with earlier paranoid delusion and content of auditory hallucination changed over time. He was admitted couple of times in the last five years due to refusal of food and becoming violent towards the family members. He was hospitalized and the psychiatrist treated him with haloperidol up to 30 mg for two months in 2010-2011. Whenever, his symptoms subsided he stopped medication. As a result the symptoms reappeared in 2012 the psychiatrist again started the haloperidol. However, this time it was ineffective.

He responded with olanzapine 20 mg. However, he discontinued the medication again. Unfortunately, family had to keep him in chain when symptoms increased. However, he was static for the few weeks until the last unexpected event occurred with his sister in law.

He tried to enjoy sex with her while she was sleeping just before the night of admission. The poor woman woke up, shouted, and escaped from him. After the incident, he attempted to commit suicide by hanging. However, his father noticed and recovered and hospitalized him. At the time of admission, he was unkempt, untidy, and perplexed. He was responding to hallucinatory voices by shouting “go away” and murmuring most of the time. His mental state examination revealed second person auditory hallucination and paranoid delusions and delusion of jealousy regarding his wife. He was changing topics frequently, sometimes in the middle of a sentence, and there was no logical connection between the different topics. His Brief Psychiatric Rating Scale (BPRS) score was 71. In the subsequent interview he confessed about sexual relation with his biological daughter. The daughter was shy, timid, and anxious; the girl tried to avoid him and she became shy and fearful. The family members noticed his behaviors few weeks before the incident occurred and tried to keep it concealed. They restricted him going close to the girl alone. Unfortunately, the family refused any psychiatric assessment of the girl. The patient was treated with risperidone and the dose was titrated to 10 mg/day by mouth. Within 3 weeks of this dose, he began to experience a decrease in his auditory hallucinations and paranoid ideation. The family was informed about the risk of further sexual abuse of the children and how they could help both the father and child for a better life. They decided to send the girl to her maternal uncle's home to study and to keep her out of his reach. However, the family refused to involve any government agencies to retain the dignity of family. Community mental health is not established in Bangladesh and lack of mental professionals with poor interagency cooperation is the main obstacle for followup and managing forensic psychiatric issues. He was discharged from the hospital 7 weeks after the admission and at the time of discharge his BPRS score decline to 32. He was brought to followup every month and his symptoms gradually declined over the two year.

## 3. Discussion

I was interested to report the case as it unfolded the neglected and hidden sexual behavioral problem of a patient suffering from schizophrenia who was also a victim of childhood sexual abuse. Sexual dysfunction is common among the people with schizophrenia [9] and around half of all patients face this problem [10]. Moreover, few researchers consider that schizophrenia is associated with aggression and violence and in most of the cases the family members are the primary victims of the violence [11]. Aggression and violence are more common when provocation from delusion and hallucination persists; the concurrent substance abuse and personality disorder or history of previous violent or antisocial behavior increase the risk [6]. The patients who suffer from schizophrenia may be perpetrator of violence; unfortunately, these patients are more commonly to become the victim of sexual violence. However, the existing evidence is inconclusive in defining whether sexual dysfunction increases the risk of violence. A complex relationship exists between schizophrenia and sexually offensive behavior [12]. The role of childhood sexual abuse in shaping up sexual behavior is well documented; however, its relation with incestuous behavior has not been explored yet. The childhood sexual abuse may predispose the patient to develop Sexual Dysfunction and Schizophrenia.

Mental illness never get adequate attention from policy makers in the low and middle income countries. Thousands of patients suffering from schizophrenia are left unnoticed, untreated, or nonadherent to treatment in Bangladesh. The nonadherence of this patient shows the importance of launching Community Psychiatry Service and the Psychiatric Social Service in the country. Most of the families try to conceal the incidents of incest [13]. The families try to conceal the incidents within themselves to avoid social discrimination, stigma, and punishment. We need to address this hidden public health emergency to increase the awareness and secure family environment. The psychiatrists should ask about sexual history including the incestuous behavior among the patients of schizophrenia in this area, overcoming the barrier of cultural shyness on sex. The superiority of risperidone in management of incestuous behavior is yet to be proven. Future researcher can explore the issue.

## 4. Conclusion

The information of occurring incest in case of schizophrenia will move the doctors to explore these neglected issues in most of the cases. To prevent this type of unwanted and undesired events the physicians need to step forward breaking the silence and cultural stigma. A regular training for the doctors, central reporting, and increasing awareness will be helpful. A holistic multisectorial approach can make every home a safe place for children.
